# Dr. Charles E. Mullins—January 15, 1932, to November 17, 2024

**DOI:** 10.1016/j.jscai.2024.102521

**Published:** 2025-01-09

**Authors:** Frank F. Ing

**Affiliations:** Division of Cardiology, University of California, Davis Children’s Hospital, Sacramento, California

**Figure 1:**
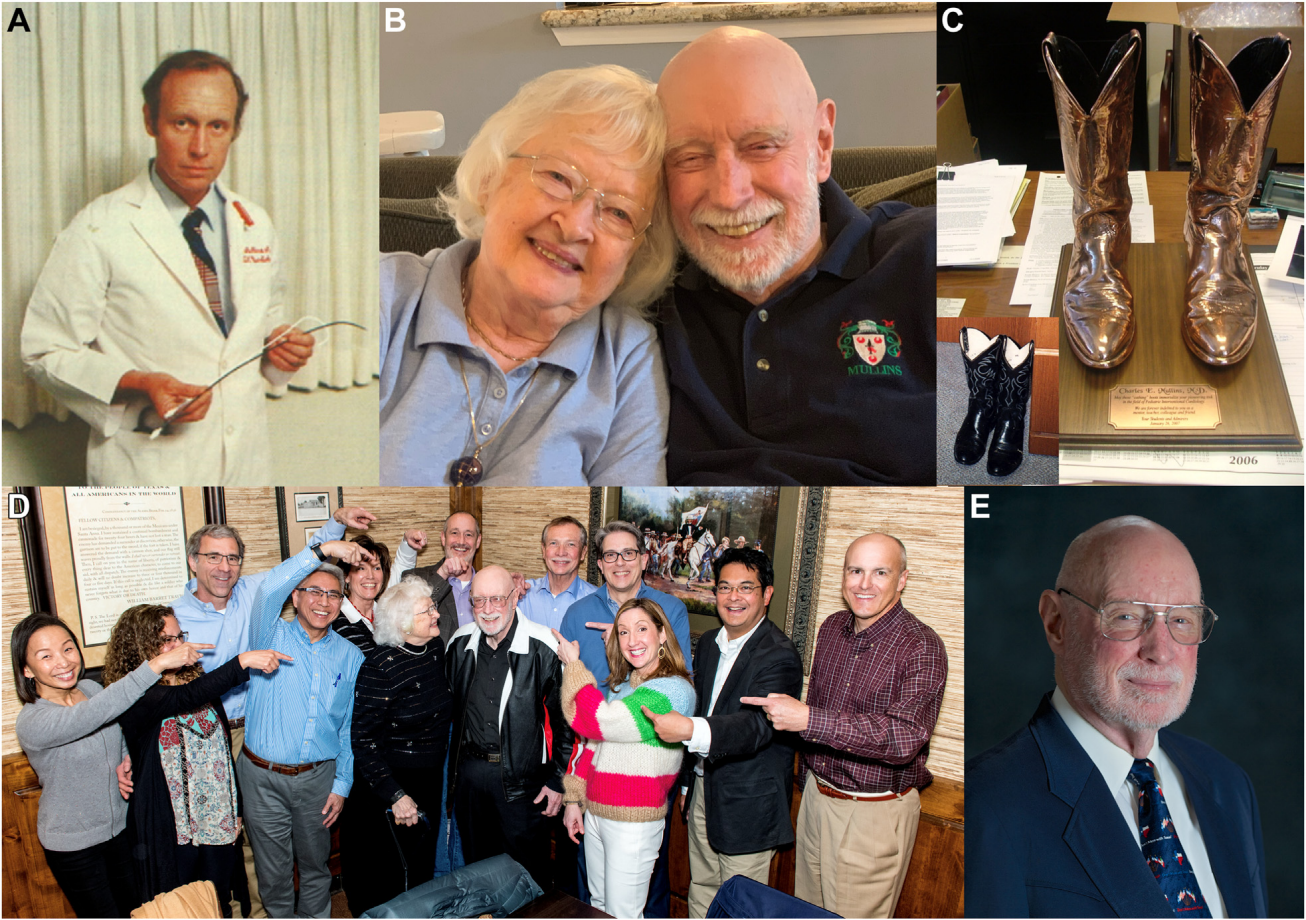
**Chuck Mullins through the years.** (A) Young Chuck with his transseptal sheath; (B) with his bride of 70 years, Arleen; (C) fellows’ gift for Chuck’s retirement—bronzed “cathing boots” stolen from his office; (D) former fellows surprise Chuck for his birthday in January 2019; (E) senior Chuck.

Dr. Charles E. Mullins, commonly known as Chuck, passed away peacefully on November 17, 2024, in Houston. News of his passing spread rapidly to many of his former trainees, colleagues, and friends who were touched deeply by his teachings and his friendship. Widely regarded as the “father” of modern pediatric interventional cardiology, Chuck had an unparalleled impact on the pediatric interventional cardiology community worldwide (Figure 1).

Not only was Chuck one of the founding fathers of the pediatric interventional cardiology fellowship back in the late 1980s, but he also contributed greatly to our field, with many novel interventional techniques and protocols for infants and children that continue to be practiced worldwide today. His contributions to device designs and trials including stents and all types of atrial septal defect, patent ductus arteriosus, and ventricular septal defect occluders propelled the pediatric intervention community into its “Golden Era” of the past 4 decades. The famous “Mullins sheath” is used worldwide for prograde left heart catheterizations and interventions. In 2023, the Palmaz Genesis XD stent was aptly rebranded by Cordis as the “Palmaz Mullins” stent in honor of him.

Chuck trained more than 150 interventional fellows and taught colleagues in more than 150 medical centers in 23 countries. He authored over 200 manuscripts and numerous book chapters. He co-authored the book *Congenital Heart Disease: A Diagrammatic Atlas* in 1988 with 167 illustrations of various congenital heart lesions.^1^ These diagrams, affectionately known as the “Mullins diagrams,” have become the gold standard for present-day congenital cardiac cath diagrams used to teach patients and students in congenital heart disease around the world. Chuck’s extraordinary passion to teach and share his knowledge was exemplified in the book’s copyright declaration, which stipulated that all the content and diagrams can be copied and used freely for teaching and training by the readership.

In 2006, after his retirement, he published a single-author 944-page book, *Cardiac Catheterization in Congenital Heart Disease: Pediatric and Adult*, in which he meticulously described the techniques of cardiac catheterization in congenital heart disease.^2^ His pioneering work and dedication to our field was recognized through numerous awards, including the inaugural Lifetime Achievement Award from the Pediatric Interventional Cardiology Symposium (PICS), the inaugural Helping Little Hearts Lifetime Achievement Award from the Society for Cardiovascular Angiography & Interventions (SCAI), as well as the Gifted Teacher Award from the American College of Cardiology (ACC) and the Founder’s Award from the American Academic of Pediatrics (AAP). However, if you asked Chuck what his greatest contribution to the field was, he would say, “the fellows I’ve trained.” He took much pride in those whom he trained and whose careers he guided over his lifetime. The relationships he cultivated with his former trainees were lifelong, as mentorship evolved into friendship. It has been a common sight to see a crowd of former students and admirers gather around Chuck after he gave a lecture at a conference. In his retirement, many of his former fellows continued to meet and enjoy a meal and conversation with him. Many visited him in Houston.

Chuck was a man of humility and took pleasure in simple things in life. He was well known for his favorite “3 Bs”: beer, (dirt) bikes, and boots. He would always choose a beer over any expensive wine during a meal. He loved to ride his dirt bike with his family at “the Patch,” a property he purchased for its hills where they can ride together. And how he loved his boots. He would always say, “they were the most comfortable footwear for the cath lab.” At his retirement, we stole his cath boots from his office and had them bronzed as a retirement gift with the inscription “May these ‘cathing’ boots immortalize your pioneering work in the field of Pediatric Interventional Cardiology. We are forever indebted to you as a mentor, teacher, colleague and friend. Your Students and Admirers, January 26, 2007.”

In his passing, we have truly lost a generational superstar pediatric interventionist, but his legacy will not only continue through his lectures, pearls of wisdom, his cath techniques, and protocols, but also through the laughter of many of his stories and “Mullins-isms” shared among his former trainees and friends, especially with a can of beer in hand at a gathering. May Chuck rest in peace. He will be missed deeply but never forgotten.

Chuck is survived by his bride of 70 years, Arleen, 3 children (Sandie, Chuck Jr, and Bob), 4 grandchildren, 3 step-grandchildren, and 5 great-grandchildren.
